# Clinical and practical considerations in the design of appropriate compensation schedules following treatment interruptions

**DOI:** 10.1259/bjro.20200041

**Published:** 2020-12-11

**Authors:** Bleddyn Jones, RG Dale

**Affiliations:** 1 Department of Oncology, University of Oxford, Gray Laboratory, Oxford, UK; 2 Green Templeton College, University of Oxford, Oxford, UK

## Abstract

Compensatory dose calculations to mitigate the deleterious effect of unscheduled treatment interruptions remain important. They may be increasingly required during and after epidemics, as with the present Covid-19 virus. The information presented to those involved in the actual dose estimations is often limited, thereby increasing the likelihood of confusion, further time delays and possibly incorrect decisions. This article sets out what aspects need to be considered by the Clinical Oncologist (or Radiation Oncologist), and the reasons why, in order to provide greater clarity. The key issues are: (a) the biological nature of the tumour (and hence its repopulation potential), (b) patient age and pre-existing medical risk factors that influence radiation tolerance, the use of chemotherapy, surgery etc, (c) the acceptable dose limits of the relevant normal tissues at risk and (d) consideration of the possibility of further field size adjustments, a change in treatment plan or acceptance of a greater role for alternative forms of radiation treatment (*e.g.* brachytherapy, electron boosts, etc.) or reliance on radical surgery. Only then can a compensatory schedule be more safely estimated.

## Introduction

There is a general consensus that unscheduled radiotherapy treatment interruptions can reduce tumour control rates and that compensatory doses are required for the more rapidly growing tumour types. Summaries of the clinical relevance and problematic nature of unintended treatment interruptions, with examples of how modifications to treatment can be made, are available in a Royal College of Radiologists Report.^1^ More general guidance for clinical teams managing cancer patients in the UK and around the world during the present coronavirus epidemic are also available on the Royal College of Radiologists website.^2^ Various teaching articles also exist, and which explain the principles and some containing worked examples and typical parameters for use within the calculations.^3–5^

The present authors have noticed considerable variation in the clinical information content when individual problems are referred for compensatory dose calculations. Important omissions can result in confusion and wasted time.

In deciding the approach to be taken for the assessment of a compensation schedule, the most relevant clinical information needs to be carefully considered before proceeding to the specific calculations, since any form of dose escalation is likely to be accompanied by increased risk of normal tissue complications. Radiation tolerances can depend, *e.g.* on age, previous and existing medical conditions, the use of surgery in the anatomical vicinity of the treatment, previous and concomitant exposure to cytotoxic chemotherapy, and it is necessary to know if the patient is already at increased risk due to such factors. In each individual case, the responsible oncologist should use his/her knowledge of the patient, coupled with some judgement, to make clear to others involved in the calculations what is the clinical intent of any derived compensation schedule and the associated relevant clinical information that may influence the choice of parameters. However, in the experience of the authors, it is often the case that compensation schedules are derived when only a minimum of relevant clinical information has been provided.

The purpose of this communication is to outline the clinical and practical aspects which should be considered by medical staff prior to conducting compensation calculations. It is emphasised that the principal aim of this process is to provide an assessment of the overall clinical status of the patient following a treatment interruption and how much, if any, relaxation of the usual normal tissue dose constraints can be allowed as part of the compensatory measures. This is necessary as compensation calculations should always use any revision to the allowable normal tissue tolerance as a starting point for devising the post-gap dose-fractionation arrangements. The uncertainties in normal tissue parameter values are generally less than those for tumours and the value of this recommended approach is that it allows at least a partial restoration of the intended tumour effect whilst minimising the likelihood that the chosen compensation schemes will result in unexpected complications.

The list below summarise the main influencing factors and it is suggested that the Consultant responsible for the treatment should assess which are likely to be most relevant in each individual case.

### Dose, time and risk factors

1. Treatment start date, treatment interruption date, and if treatment has been resumed already, or the expected date of treatment resumption.
2. Prescribed dose and fractionation.
3. Organ at risk (OAR) percentage doses in the original treatment plan.
4. The expectation of risk if the treatment had been delivered as per prescription.

### Clinical and patient factors

1. Age.
2. Relevant past medical history.
3. Treatment intent (radical, palliative,preoperative, or post-operative and if so when was surgery done).
4. Tumour details: histopathology diagnosis, grade and stage with any features that may indicate slower or faster growth kinetics.
5. Other treatments (surgery, cytotoxic chemotherapy and any other form of anticancer treatment, *e.g.* Cetuximab or other biological modifiers.
6. How treatment was tolerated before the interruption?
7. Reason for interruption (due to normal tissue reaction, intercurrent illness or other cause).
8. Previous radiotherapy courses in the same anatomical region if relevant.
9. Has further tumour imaging and treatment planning already been conducted, and, if not, is this possible?
10. Have the OAR doses changed if a new treatment plan has been done?

### Logistical factors

1. The scope for further treatment using two fractions per day.
2. Are weekend or partial weekend treatments (even if only one per day) possible?

In most radiotherapy departments, it is likely that Physicists will calculate gap corrections and, although they might appreciate the relevance of the tumour histology, they could not be expected to know how aspects such as concomitant health issues, concurrent chemotherapy, surgical issues, etc can impact on overall radiation tolerance. In this respect, the above list sets out the clinical and practical points (most of which will be well-known to the responsible clinician) which should inform the decision on what the treatment compensation is required to achieve whilst taking account of any altered balance between tumour control (*i.e.* maintained as prescribed or with an allowed reduction) and a possibly increased risk of toxicity. Once the objective of the compensation has been agreed the clinician and physicist (or other individual responsible for calculating gap compensations) can together consider possible scheduling patterns which will achieve (or closely approach) the desired clinical aim.

In some cases, clinicians may find it helpful to express their requirement (having considered the clinical status of the patient) in terms of a maximum-allowable EQD2 for the OARs, *i.e*. the maximum normal tissue dose the clinician would allow in a complete schedule if it were delivered in 2 Gy fractions. Such information can facilitate calculation of compensation schemes which involve other fractionation patterns designed to maintain the maximum-allowable normal tissue EQD2 whilst allowing at least some restoration of tumour effect.

The compensatory arrangements should also consider, where practicable, possible changes to the physical aspects of treatment delivery, *e.g*. altered field sizes or beam numbers. For longer interruptions, the tumour and normal tissue anatomical relationships may themselves change, bringing possibilities of reduced treatment volumes for the remainder of treatment, or even revision to the overall treatment policy.

If local compensation expertise is not available, then advice needs to be obtained elsewhere, *e.g*. from an expert advisory panel consisting of clinicians and physicists. As discussed above, the clinical requirements would first need to be condensed by the responsible local clinician into a form which a remote physicist adviser could handle, primarily providing an assessment of the extent to which the normal tissue dose can be increased, what altered fractionation patterns are feasible, any local time constraints, etc. If the query is because the responsible clinician is seeking advice on the relevance of some aspect of the medical history, then that question must go direct to a fellow clinician.

In all cases, the consultant carries the ultimate medicolegal responsibility for the administration of compensatory treatments and their possible consequences and it is therefore essential that formal consenting of the altered treatment is obtained.

### The national position

There is no current structure for ensuring adequate consistency of advice across the four nations within the UK National Health Service. Such a deficit needs to be addressed by the professional bodies concerned with radiotherapy as well as by each of the Health Services. A trans-UK service for advice would be a reasonable goal for especially difficult individual problems and in times of crisis. During the Covid-19 outbreak, an *ad hoc* group of persons with experience of such estimations was formed and provided updated advice to the Royal College of Radiologists and the Institute of Physics and Engineering in Medicine. Several individuals in that small group are retired and no longer in possession of medical indemnity, so longer term arrangements need to be put in place. The importance of practical experience in performing compensation calculations cannot be underestimated and the recent Royal College of Radiologists report has highlighted the need for appropriate training courses^1^ to help develop the nationally available skill set. A national reporting system of treatment interruptions would also provide a data base for future analysis, especially since clinical trials would be difficult to conduct.

It is hoped that this short article will set a standard for treatment compensation referrals.
